# Lifetime Stress Cumulatively Programs Brain Transcriptome and Impedes Stroke Recovery: Benefit of Sensory Stimulation

**DOI:** 10.1371/journal.pone.0092130

**Published:** 2014-03-20

**Authors:** Fabíola C. R. Zucchi, Youli Yao, Yaroslav Ilnytskyy, Jerrah C. Robbins, Nasrin Soltanpour, Igor Kovalchuk, Olga Kovalchuk, Gerlinde A. S. Metz

**Affiliations:** 1 Canadian Centre for Behavioural Neuroscience, University of Lethbridge, Lethbridge, Alberta, Canada; 2 Departments of Medicine and Biological Sciences, University of Mato Grosso State, Cáceres, MT, Brazil; 3 Department of Biological Sciences, University of Lethbridge, Lethbridge, Alberta, Canada; University of Tennessee, United States of America

## Abstract

Prenatal stress (PS) represents a critical variable affecting lifetime health trajectories, metabolic and vascular functions. Beneficial experiences may attenuate the effects of PS and its programming of health outcomes in later life. Here we investigated in a rat model (1) if PS modulates recovery following cortical ischemia in adulthood; (2) if a second hit by adult stress (AS) exaggerates stress responses and ischemic damage; and (3) if tactile stimulation (TS) attenuates the cumulative effects of PS and AS. Prenatally stressed and non-stressed adult male rats underwent focal ischemic motor cortex lesion and were tested in skilled reaching and skilled walking tasks. Two groups of rats experienced recurrent restraint stress in adulthood and one of these groups also underwent daily TS therapy. Animals that experienced both PS and AS displayed the most severe motor disabilities after lesion. By contrast, TS promoted recovery from ischemic lesion and reduced hypothalamic-pituitary-adrenal axis activity. The data also showed that cumulative effects of adverse and beneficial lifespan experiences interact with disease outcomes and brain plasticity through the modulation of gene expression. Microarray analysis of the lesion motor cortex revealed that cumulative PS and AS interact with genes related to growth factors and transcription factors, which were not affected by PS or lesion alone. TS in PS+AS animals reverted these changes, suggesting a critical role for these factors in activity-dependent motor cortical reorganization after ischemic lesion. These findings suggest that beneficial experience later in life can moderate adverse consequences of early programming to improve cerebrovascular health.

## Introduction

The perinatal environment is critical for programming long-term physiology and health. Animal studies have shown that an early adverse environment can reprogram activity of the hypothalamic-pituitary-adrenal (HPA) axis and stress responsiveness [1]-[3], behaviour and mental health [4]–[6] and elevate the risk of metabolic and cardiovascular disease, such as hypertension [7]–[8]. Given the positive association between hypertension and stroke risk [9], programming of the HPA axis by perinatal stress may critically influence stroke risk and outcome in adulthood. Furthermore, elevated HPA axis activation by stress in adulthood has been shown to diminish motor recovery after ischemic lesion in a rat model [10]–[12] and synergistically impair outcomes in aged rats [13].

Although the early environment sets the stage for adult health outcomes, experience throughout the life span may modify the imprints of perinatal programming. Experiential therapies have been shown to effectively promote recovery from stroke, including environmental enrichment [14] and tactile stimulation [15]–[16] and its equivalent in humans, massage therapy [17]. TS may ameliorate elevated HPA axis activity [18]–[20], and modulate the expression of genes related to immune functions [21]. Furthermore, TS may alleviate the effects of early adverse experience in humans and animals [22]–[24]. Morphological and neurochemical manifestations of TS may include elevated neurotrophic factor expression, facilitated cortical rearrangement and synaptogenesis [15], [25], [16]. These observations suggest that TS, through neuroprotective pathways that involve mRNA expression of neurotrophic and synaptogenic factors, may also promote recovery from an ischemic lesion.

Here we investigated the influence of prenatal stress on focal ischemia outcomes and the benefit of TS in a rat model of stroke. We hypothesized that enriching the environment with TS may reduce the effects of adverse perinatal experience in healthy animals, and facilitate recovery from ischemic cortical damage in adulthood. We show that prenatal stress programs the adult stress response and compromises behavioural and structural recovery from ischemic brain damage in association with differential gene expression profiles. We also show that cumulative effects of stressful experiences throughout prenatal and postnatal periods affect the transcription of genes related to central pathways of neuronal survival and plasticity. Lastly, we show that TS counteracts the detrimental effects of chronic stress across prenatal and postnatal periods and facilitates recovery from ischemic brain injury through altered gene expression and enhanced motor recovery. Thus, this study demonstrates a link between perinatal programming by adverse environment and cerebrovascular health later in life.

## Materials and Methods

### Ethics Statement

All procedures were performed in accordance with the guidelines of the Canadian Council on Animal Care and approved by the University of Lethbridge Animal Welfare Committee.

### Experimental Design

Fourty-two male Long-Evans rats were used. Animals were raised at the local vivarium and housed in pairs under a 12 hr light/day cycle with lights on at 7:30AM. To encourage participation in the skilled reaching task, animals underwent a mild restricted feeding schedule to maintain body weight at 90–95% of baseline weight to motivate participation in the skilled reaching task. After completion of the first five training days, animals were allowed to gain weight.

**Figure 1:**
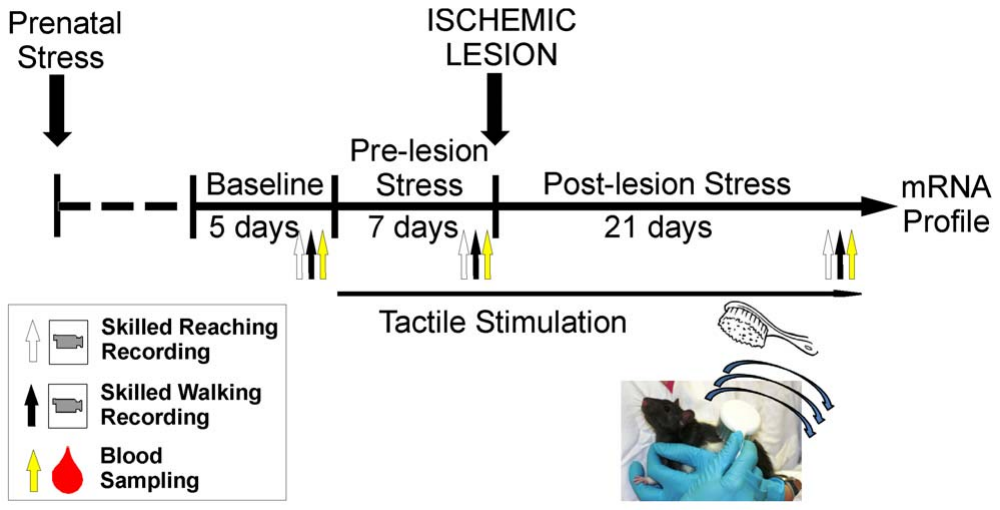
Experimental design. Time-course of procedures. Adult male offspring was pre-trained and tested in a pellet reaching task and skilled walking. Blood samples were collected three times for corticosterone assessments. Stress and TS were induced for four weeks. Transcriptomic analyses were performed after behavioural testing was completed.

The time course of manipulations is shown in Figure 1. Prenatally stressed and non- stressed male rats were assessed in a behavioural test battery in adulthood. At 90 days of age rats were trained in the skilled reaching task until success rates reached an asymptote (3 weeks). The next five days of skilled reaching were considered for baseline measurements and performance was videorecorded on day six for quantitative analysis. Rats were also pre-trained and videorecorded in the skilled walking task. On days without behavioural testing, blood samples were collected for corticosterone (CORT) assessments. One to two male offspring per litter were assigned to groups of combinations of prenatal stress (PS), adult stress (AS), tactile stimulation (TS), and ischemic lesion (Lx): 1) PS+Lx (n = 8); 2) PS+AS+Lx (n = 8); 3) PS+AS+TS+Lx (n = 8). Controls were assigned to: 1) ischemic lesion Lx (n = 9); 2) non-lesion Control (n = 9). AS by restraint stress and TS were given for ten days prior to the lesion, after which behaviour was videorecorded again. Behavioural testing, AS and TS continued until three weeks post-lesion (i.e., weeks 1, 2 and 3 after lesion, Figure 1) and final videorecordings and blood samples were collected. After completion of tests, rats were sacrificed and brains and adrenal glands were collected for physiological and molecular assessments. Motor cortices from lesion and non-lesion hemispheres were extracted for comparison of global gene expression between hemispheres and between treatments.

### Ischemic Motor Cortex Lesion

Rats were anesthetized using isoflurane in an oxygen/nitrous oxide mixture (isoflurane 4% for initiation, 2% for maintenance at an oxygen flow rate of 2.0 l/min). A focal lesion of the motor cortex contralateral to the paw preferred in skilled reaching was induced via devascularization [26], [11]–[12]. Briefly, the skin over the skull was incised and the skull was exposed. Using a fine dental burr, a craniotomy was made at the following coordinates: –1.0 to 4.0 mm anterior-posterior to Bregma and 1.5 to 4.5 mm lateral to Bregma. This area is partially supplied by the middle cerebral artery [27]. The dura and the blood vessels were carefully removed by gently rubbing the superficial blood vessels with a cotton tip that was soaked in saline solution. This procedure removed the distal branches of the middle cerebral artery (MCA) and of the anterior cerebral artery (ACA), as well as any minor blood vessels. Then the skin was sutured and the rat was given analgesic (Temgesic, Schering-Plough, Brussels) and placed on a heating pad until fully awake.

### Stress Paradigms

#### Prenatal

Timed-pregnant rats were stressed twice daily from gestational days 12 to 18. Two stressors, restraint of the body for 20 min in a transparent Plexiglass cylinder [28], [12] and forced swimming in water at room temperature for 5 min [29]–[30] were given daily in alternating intervals in the morning and afternoon hours.

#### Adult

Stress in adulthood was induced by restraining animals in a standing position in a transparent Plexiglass cylinder for 20 min daily [28], [12].

### Tactile Stimulation

TS was induced by handling animals and stroking their back with a soft baby hair brush for 20 min daily [31] after behavioural testing was completed. All non-TS groups received handling by the experimenter.

### Behavioural Training and Testing

#### Skilled reaching

Skilled forelimb function was assessed in a skilled pellet reaching task as described earlier [32]–[33]. In each training and test session, rats were placed individually in a reaching box and a food pellet (45 mg each, BioServ, Frenchtown, NJ) was placed contralaterally to the rats’ preferred reaching paw. To readjust their body position, rats were trained to walk to the rear end of the box before reaching for a new pellet. Each rat was given 20 pellets per training and test session. A successful reach was defined as obtaining the pellet on the first attempt, withdrawing the paw through the slit and releasing the pellet to the mouth. The percentage of successful reaches and the number of pellets obtained were calculated [33]. To assess reaching accuracy, the number of attempts to grasp a single pellet was averaged.

Qualitative analysis of skilled reaching trajectories was performed from videorecordings using frame-by-frame video inspection by a blind observer. Reaching movements were broken down into eleven components and 35 subcomponents as described by Metz and Whishaw [33]. Each of the subcomponents was scored on a 3-point scale. A score of 1 was given if the movement was present. A score of 0.5 was given if the movement was present but abnormal, and a score of 0 was given if the movement was absent. An average score for each component was calculated based on three successful reaches.

#### Skilled walking

Fore- and hindlimb coordination and limb placement were assessed using the ladder rung walking task [34]–[35]. An irregular pattern of rungs was used to prevent learning effects in repeated test sessions. No further reinforcement was given to motivate the animals to cross the ladder. Animals were trained or tested three times per session. Qualitative analysis was performed by frame-by-frame analysis by a blind observer according to Metz and Whishaw [34]. The types of foot or paw placement on the rungs were rated using a 7-category scale with 6 representing optimum performance and 0 representing failed steps.

### Corticosterone Assays

Blood samples were taken from the tail vein (0.6 ml) 10 minute post-stress under 4% isoflurane anesthesia [28]. Blood samples were collected between the hours of 9:10–10:30 AM. No behavioural testing was performed on blood sampling days. Plasma CORT concentrations were determined by ELISA (Corticosterone Enzyme Immunoassay Kit, Cayman Chemical Company, Ann Arbor, MI).

### Postmortem Tissue Collection

Animals received an overdose of pentobarbital (Euthansol 100 mg/kg; CDMV Inc., Québec, Canada) via intraperitoneal injection. Rats were rapidly decapitated for tissue collection and the motor cortex from the lesion hemisphere and its contra-lateral hemisphere was dissected and flash frozen for transcriptomic analysis. The adrenal glands were removed and weighed. Right and left adrenal glands weight was averaged.

### Global Gene Expression

#### RNA preparation and microarray analysis

Total RNA was extracted from the motor cortex lesion and non-lesion hemispheres in three rats per group. TRI Reagent Solution (Applied Biosystems, Foster City, CA) was used according to the supplier’s protocol. RNA was purified using the RNeasy total RNA cleanup protocol (Qiagen, Hilden, Germany). Quality control of the RNA samples was performed using Bioanalyzer Eukaryote Total RNA Nano Chip (Agilent, Santa Clara, CA). Microarray analysis (probe synthesis, hybridization, and scanning) was performed using a standard Illumina platform protocol by Genome Quebec. All statistical analyses were performed considering both group effect and lesion effect. Group effects considered the following variables: prenatal stress (group PS+Lx vs. group Lx); cumulative prenatal and adult stress (group PS+AS+Lx vs. group PS+Lx); ischemic lesion (lesion Lx group vs. non-lesion naive control); TS (group PS+AS+TS+Lx Vs. group PS+AS+Lx). The lesion effect was considered between lesion and non-lesion hemispheres in each group (lesion vs. non-lesion hemispheres). Raw data were adjusted for background and normalized using the Robust Microchip Average (RMA) method [36]. Statistical analyses were performed with two-way ANOVA (variables: group and lesion) using FlexArray 1.5 (Genome Quebec, Montreal, QC). Transcripts were defined as significantly differentially expressed if the expression levels showed a fold change of ≥2 with a *p*-value of ≤0.05, and data were presented as log2. Genes commonly expressed among treatments and genes modulated by treatment were determined. Differentially expressed probes were classified according to GeneOntology terms (GeneOntology allows classification of genes according to their molecular function, biological process, cellular component, and chromosomal localization).

#### Quantitative Real-Time PCR

In order to validate gene expression patterns, we performed qRT-PCR analysis of eleven differentially regulated genes [37]. The following mRNAs were analyzed: *Lgals3*, *S100a4*, *Vim*, *B2m*, *Egr1*, *F2r*, *Fabp7*, *Gfap*, *Hmgn*, and *Gst3* (Table 1). The inclusion criteria for genes used for RT-PCR validation were higher frequency and higher expression levels of genes commonly expressed, and genes differentially expressed by treatment. The same samples of total RNA used for microarray analysis were used for qPCR analysis. Total RNA samples cleanup process was performed using Illustra RNAspin Mini RNA Isolation Kit (GE Healthcare, Wilmington, MA). The generation of cDNAs from the total RNA samples was performed using RevertAid™H Minus M-MuLV Reverse Transcriptase First Strand cDNA Synthesis Kit #K1631, #K1632 (Thermo Fisher Scientific Inc, Marietta, OH). Oligo DT primers were used for obtaining mRNA present in the samples. RT-PCR reactions were performed using Bio-Rad CFX96™ Real-Time PCR Systems with SsoFast EvaGreen Supermix (Bio-Rad, Hercules, CA) reaction cocktail added to the cDNAs templates and specific primers. A total volume of 20 μl was used, with 1 μl of cDNA template, 400 nM forward primer, 400 nM reverse primer, and 10 μl of SsoFast EvaGreen Supermix (Bio-Rad, Hercules, CA). PCR-product amplification was performed in three technical repeats per experiment, which was confirmed by analysis using 1% agarose electrophoresis gels. All measurements were compared to naïve controls values. *Gadph* was used as a reference control for the calculation of gene expression ratio. Means of gene expression were compared to *Gadph* expression. Analysis of variance (one-way ANOVA) was performed to identify the selected gene expression levels among treatment groups and brain hemisphere using Bio Rad CFX Manager v2.0 [38].

**Table 1:** Primers used for qRT-PCR gene expression profile validation.

*Gene*	*Forward primer*	*Reverse primer*
***VIM***	ATGTTGACAATGCTTCTC	CTCCTGGATCTCTTCATC
***S100A4***	TCCTCAGATGAAGTGTTG	TATGAAGAAGCCAGAGTAAG
***LGALS3***	TCACAATCATAGGCACAGT	TAAAGTGGAAGGCGATGT
***HMGN1***	GTGATAATGTGCTGTGAA	CCTTACGACCTCTCATAA
***GST3***	CTCTAAGGAATATGGATT	TCTATCTTGTACTTCTTG
***GFAP***	TGGGTTAGAACTGGAAGA	AACAACAAGGATGAAGGAA
***FABP7***	CACATTCAAGAACACAGA	CGAATCACAGACTTACAG
***F2R***	CTGAGAGGATGTATGCTA	TGTATCTTCACTGGGATT
***EGR1***	TCCACTATCCACTATCAA	ACTGGTAGGTGTTATTAAG
***B2M***	CAACTTCCTCAACTGCTA	GGTATCTTCTTTCCATTCTTC
***GADPH***	CATTCTTCCACCTTTGAT	CTGTAGCCATATTCATTGT

### Statistical Analysis

Behavioural and physiological data were analysed by analyses of variance (ANOVA), unpaired (between-group comparisons), and paired Student’s t-tests (within-group comparisons) using Statview software version 5.0 (SAS Institute, 1998). Data are presented as means±SEM, and asterisks indicate significances as follows: *p < 0.05; **p < 0.01; ***p < 0.001.

The gene datasets were analyzed using FlexArray software developed by M. Blazejczyk and associates (Genome Quebec, Montreal, QC). In brief, all statistical analyses were performed considering both group effect and lesion effects (details in *RNA preparation and microarray analysis*). Raw data were adjusted for background and normalized using the Robust Microchip Average (RMA) method [36]. To identify sets of differentially regulated genes among groups, analysis of variance was performed (two-way ANOVA; variables: group and lesion) using FlexArray 1.5 (Genome Quebec, Montreal, QC). The cut-off was a 2-fold change and *p*<0.05.

PCR-product amplification was performed in three technical repeats per experiment, and was confirmed by analysis using 1% agarose electrophoresis gels. All measurements were compared to naïve control values. *Gadph* was used as a reference control for calculation of gene expression ratio. Means of gene expression were compared with *Gadph* expression. Analysis of variance (one-way ANOVA) was performed to identify the selected genes expression levels among treatment groups and brain hemisphere, using Bio Rad CFX Manager v2.0 [38].

## Results

### Stress-Induced Reduction in Skilled Movement Success is Alleviated by Tactile Stimulation

**Figure 2:**
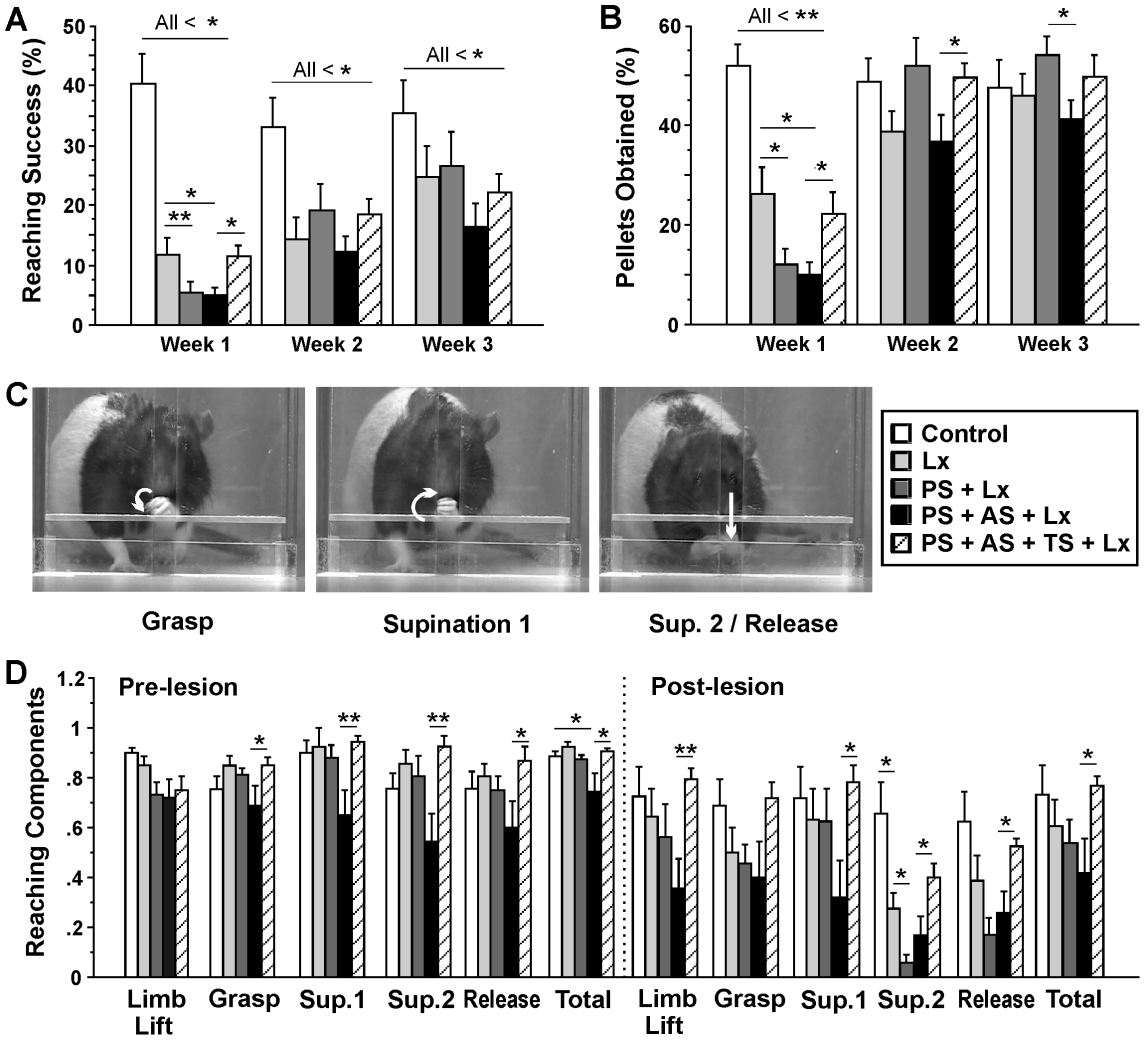
Tactile stimulation restored forelimb function after ischemic lesion. **A,** Success rate in the single pellet reaching task; **B,** Number of pellets obtained; **C,** Sequence of video frames depicting grasp, supination 1 and 2, and release components; **D,** Qualitative assessment of reaching components. Note that the ischemic lesion reduced quantitative (success, pellets obtained) and qualitative aspects of skilled reaching. Cumulative effects of prenatal and adult stress further reduced skilled reaching ability. TS promoted reaching success and movement ability. *p<0.05, **p<0.01.

Overall, there were significant differences in success rates among groups (F(4,38) = 3.81, p<0.001). While there were no pre-lesion group differences, the ischemic lesion reduced reaching success in the first and second weeks after lesion compared to non-lesion controls (t(16) = 4.99, p<0.001; t(16) = 3.00, p<0.01, respectively; Figure 2A). Success rates significantly improved in post-lesion weeks 2 and week 3 compared to week 1 (t(8) = 5.55, p<0.001; t(8) =  –4.32, p<0.01, respectively). Initially after lesion PS and both PS+AS reduced success rates compared to animals with lesion only (t(15) = 6.38, p<0.001; t(15) =  –2.13, p<0.05, respectively), which subsequently improved (PS: t(7) = 6.26, p<0.001; t(7) =  –3.21, p<0.05; PS+AS: t(7) = 4.42, p<0.01; t(7) =  –2.58, p<0.05, respectively; Figure 2A). Notably, TS lesion animals performed significantly better than their stressed counterparts one week after lesion (compared to PS+Lx: t(15) = 2.42, p<0.05; compared to PS+AS+Lx: t(15) =  –2.63, p<0.05, respectively) and they were not different from lesion only controls. Again, they subsequently improved (t(8) = 5.73, p<0.001; t(8) =  –4.65, p<0.01, respectively).

### Stress Diminishes Skilled Movement Accuracy and is Attenuated by Tactile Stimulation

Overall, there were significant differences in movement accuracy, as indicated by the number of pellets obtained, among groups after ischemic lesion (F(4,38) = 2.66, p<0.05; Figure 2B). All treatments reduced reaching accuracy during the first week after lesion compared with non-lesion rats (all p’s p<0.01) followed by significant improvement (all p’s<0.05). PS and PS+AS showed further reduced reaching accuracy compared to lesion-only performance (p’s<0.05). PS+AS showed reduced reaching accuracy three weeks after lesion, compared to non-lesion control (t(15) = 7.79, p<0.001), lesion only (t(15) =  –2.13, p<0.05) and PS lesion rats (t(14) =  –2.38, p<0.05). Lower reaching accuracy than non-lesion controls occurred in TS-treated and lesion-only rats only in week 1 (t(16) = 4.71, p<0.001; Figure 2B). TS-treated rats performed significantly better than PS+AS rats in the first and second weeks after lesion (t(15) =  –2.40, p<0.05; t(15) =  –2.18, p<0.05; Figure 2B). Lesioned TS-treated rats returned to non-lesion levels by week 2 as did PS rats, but not lesion-only controls or PS+AS lesion rats.

### Tactile Stimulation Protects Against Stress-induced Motor Loss

Qualitative analysis of skilled reaching movement components revealed an interaction of group and reaching components (F(4,37) = 1.46, p<0.001; Figures 2C-D). Cumulative stress in PS+AS animals decreased the total reaching score compared to controls (t(15) = 2.58, p<0.05). The most prominent pre-lesion changes were induced by TS, which reduced the PS+AS-induced motor disabilities in grasp, supination 1, supination 2, and release (all p’s<0.05). These changes were also reflected in an overall improved total reaching score (t(14) =  –2.36, p<0.05). From baseline to pre-lesion, within-animal comparison showed that TS increased supination 1 (t(8) =  –2.44, p<0.05) and 2 (t(8) =  –3.64, p<0.01), and release scores (t(8) =  –2.35, p<0.05).

Compared to controls, the lesion induced a decrease in supination 2 (t(16) = 3.23, p<0.01). Lesion PS rats displayed a reduced ability to supinate compared to lesion rats (supination 2, t(15) =  –2.83, p<0.05). Furthermore, from pre-lesion to post-lesion, PS lesion animals showed impaired ability to orient (t(7) = 2.43, p<0.05), digits close (t(7) = 2.48, p<0.05), digits open (t(7) = 2.60, p<0.05), grasp (t(7) = 4.73, p<0.01), supination 2 (t(7) = 10.89, p<0.001), and release (t(7) = 7.72, p<0.001), reflecting in a reduced total score (t(7) = 3.79, p<0.01). The lesion in the PS+AS group led to reduced limb lift (t(8) = 2.87, p<0.05), supination 2 (t(8) = 3.23, p<0.05), release (t(8) = 2.60, p<0.05), and total scores (trend: t(8) = 2.03, p = 0.0818). Again, TS significantly improved movement performance in PS+AS rats in limb lift (t(14) =  –3.22, p<0.01), supination 1 (t(14) =  –2.53, p<0.05), supination 2 (t(14) =  –2.22, p<0.05), release (t(14) =  –2.74, p<0.05), and total movement score (t(14) =  –2.26, p<0.05). However, from pre-lesion to post-lesion PS+AS+TS animals showed loss in supination 2 (t(8) = 7.92, p<0.001), release (t(8) = 5.74, p<0.001), and total movement score (t(8) = 2.99, p<0.05).

### Tactile Stimulation Promotes Restoration of Motor Control in Skilled Walking

**Figure 3:**
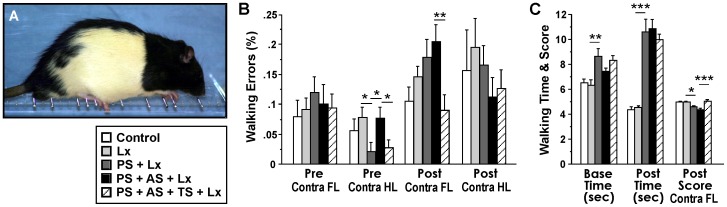
Tactile stimulation offset effects of cumulative stress in skilled walking ability. **A,** A rat performing the ladder rung walking task; **B,** Walking error rate; **C,** Walking time and score. Prenatal stress prolonged the time to cross the ladder at baseline and post-lesion periods. Cumulative stress exaggerated the effects of ischemia in limb placement and inter-limb coordination. TS reduced limb placement errors and improved time measurements and scores. *p<0.05, **p<0.01, ***p<0.001.

Figure 3B shows the group differences in limb placement errors in the ladder rung walking task (F(4,36) = 6.92, p<0.001). Pre-lesion data show that PS reduced error rates in hindlimbs corresponding to the dominant hemisphere (t(15) =  –2.48, p<0.05). Furthermore, TS reduced hindlimb error rates in PS+AS rats compared to PS treatment only (t(15) = 2.16, p<0.05).

The ischemic lesion increased forelimb error rates contralateral to the lesion hemisphere from pre- to post-lesion periods in the lesion-only group (t(8) =  –2.43, p<0.05), the PS+Lx group (t(7) =  –4.69, p<0.01) and in the PS+AS+Lx group (t(7) =  –3.54, p<0.01). Hindlimb error rates increased from pre- to post-lesion period only in the PS+AS+TS+Lx group (t(8) =  –3.10, p<0.05). Notably, TS significantly reduced the relative, non-significant post-lesion increase in forelimb errors rates in PS+AS+Lx animals (t(15) = 3.04, p<0.01; Figure 3B).

The time needed to cross the horizontal ladder was longer in PS rats than in controls at baseline and post-lesion periods (t(15) = 3.14, p<0.01; t(15) = 6.77, p<0.001, respectively; Figure 3C). There was no effect of TS on this variable.

The movement score revealed overall differences across groups in the ladder rung walking task (F(4,38) = 1.90, p<0.001; Figure 3C). Compared to pre-lesion levels, the lesion decreased fore- and hindlimb scores in PS rats (all p’s<0.01) and in PS+AS rats (all p’s<0.001) and increased time measurements in PS rats (t(7) =  –5.08, p<0.01) and PS+AS rats (t(7) =  –5.87, p<0.001). Hindlimb scores decreased in PS+AS+TS+Lx rats (t(8) = 2.83, p<0.05). Post-lesion, PS decreased movement scores in the forelimb contralateral to lesion (t(15) =  –2.65, p<0.05; Figure 3C). Notably, TS improved forelimb error scores in PS+AS+Lx animals compared to their non-treated counterparts (t(15) = 3.04, p<0.01).

### Prenatal and Cumulative Stress Exaggerates HPA Axis Activity

**Figure 4:**
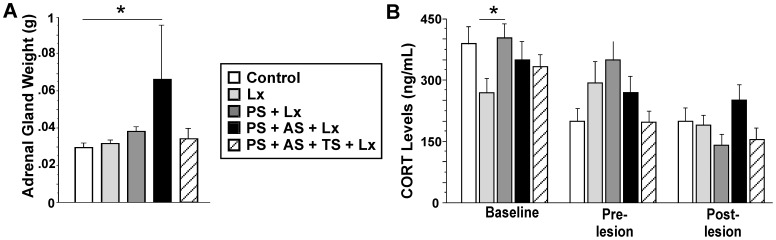
Cumulative effects of prenatal and adult stress elevated HPA axis activity. **A,** Adrenal gland weight; **B,** Plasma CORT levels. Animals stressed both prenatally and in adulthood showed larger adrenal glands and elevated CORT levels in response to the ischemic lesion. *p≤0.05.

Adrenal gland weight was higher in the PS+AS+Lx group (0.066) compared to controls (0.030; p>0.05) indicating elevated HPA axis activity (Figure 4A). Nevertheless, at the time of sampling there were no differences in CORT levels among groups (Figure 4B). However, baseline CORT levels in PS+Lx were higher than in Lesion-only rats (p<0.05), reflecting the prenatal stress effect at baseline.

### Adverse and Beneficial Experiences Differentially Alter the Motor Cortex Transcriptome

Stress-induced motor impairments may be the result of long-term reprogramming of gene expression. Thus, we used microarray analyses to determine differential transcriptomic profiles by treatment and hemispheres.

**Figure 5:**
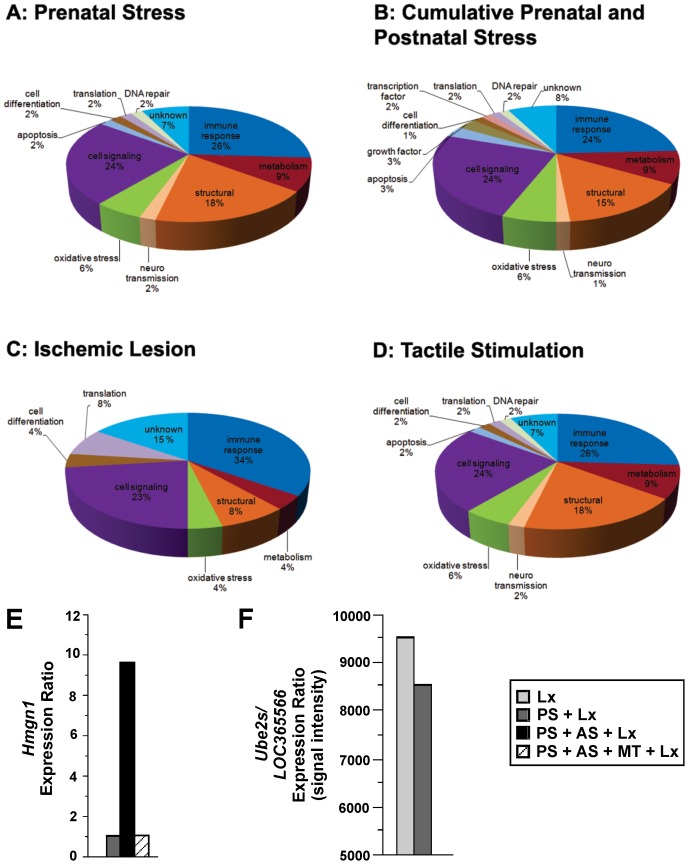
Cumulative stress and tactile stimulation differentially altered motor cortex gene expression. Microarrays of motor cortex gene expression after **A,** PS; **B,** cumulative prenatal and adult stress; **C,** ischemic lesion; **D,** TS. Note that cumulative effects of prenatal and adult stress increased the variety of expressed genes; **E,** Microarray analysis of motor cortex *Hmgn1* expression. Cumulative stress increased *Hmgn1* levels, when compared with PS+Lx group, and tactile stimulation restored expression levels; **F,** Prenatal stress down-regulated *Ube2s/LOC365566* expression.

Cross comparison analyses between treatments and brain hemispheres (lesion or non-lesion) provided mRNA expression profiling specific to prenatal stress, cumulative prenatal and adult stress, ischemic lesion and the effects of tactile stimulation (Figure 5A-D). Genes expressed were classified in 15 categories, according to their cellular function (Illumina ontology definition): neurotransmission, oxidative stress, cell signaling, apoptosis, growth factors, cell differentiation, transcription factor, translation, DNA repair, immune response, metabolism, structural maintenance, myelination, virus resistance, and unknown functions (Table 2). Cutoff criterion was based on twofold change (up- or down-regulated, presented as log2) and p<0.05 statistical significance determined genes commonly (among different treatments; Table 2) and uniquely (per treatment; Table 3) regulated.

**Table 2:** Genes commonly expressed across treatments.

*Genes*	*Category*	*Frequency*
***Hspb1***	Apoptosis	10
***Ncf1***	Apoptosis	5
***Lr8***	cell differentiation	9
***Prp19***	cell differentiation	1
***Ftl1***	cell signaling	10
***Igsf1***	cell signaling	8
***Lgals1***	cell signaling	9
***Lgals3***	cell signaling	15
***LOC305633***	cell signaling	1
***LOC498276***	cell signaling	10
***LOC498279***	cell signaling	9
***Mgp***	cell signaling	1
***Ogn***	cell signaling	6
***Rbp1***	cell signaling	12
***S100a11***	cell signaling	13
***S100a13***	cell signaling	5
***S100a4***	cell signaling	14
***S100a6***	cell signaling	12
***Smpdl3a***	cell signaling	12
***Spp1***	cell signaling	11
***Tf***	cell signaling	6
***Trem2***	cell signaling	5
***Zcchc9***	cell signaling	1
***Hmgn1***	DNA repair	4
***Bmp7***	growth factor	4
***Igf2***	growth factor	1
***Igfbp2***	growth factor	5
***Igfbp3***	growth factor	4
***A2m***	immune response	13
***Aif1***	immune response	1
***Anxa1***	immune response	12
***Anxa3***	immune response	4
***C1qa***	immune response	9
***C1qb***	immune response	9
***C1r***	immune response	6
***C1s***	immune response	7
***C2***	immune response	13
***C4-2***	immune response	6
***Cd48***	immune response	8
***Cd68***	immune response	11
***Chi3l1***	immune response	3
***F2r***	immune response	3
***Igsf7***	immune response	3
***Mgst1***	immune response	8
***Nupr1***	immune response	7
Rt1-a2	immune response	10
***Rt1-da***	immune response	9
***Serping1***	immune response	14
***Adrp***	metabolism	6
***Aebp1***	metabolism	1
***Dab2***	metabolism	1
***Fabp7***	metabolism	11
***Grn***	metabolism	11
***Gstp1***	metabolism	1
***Ifitm3***	metabolism	9
***Klk6***	metabolism	8
***Lcat***	metabolism	4
***Npc2***	metabolism	9
***Timp1***	metabolism	8
***Ttr***	metabolism	1
***Mal***	myelination	1
***Mbp***	myelination	5
***Tspan2***	myelination	1
***Bzrp***	neurotransmission	1
***Slc6a5***	neurotransmission	5
***Cyba***	oxidative stress	11
***Gpx1***	oxidative stress	4
***Mt1a***	oxidative stress	9
***Txnip***	oxidative stress	10
***Arc***	structural	3
***Cldn11***	structural	8
***Col1a2***	structural	13
***Col3a1***	structural	4
***Col6a3***	structural	7
***Dcn***	structural	1
***Emp3***	structural	10
***Fn1***	structural	4
***Gfap***	structural	15
***Gjb2***	structural	8
***Gp38***	structural	1
**Gsn**	structural	1
***Lcp1***	structural	8
***LOC292539***	structural	10
***RGD1308367***	structural	1
***Vim***	structural	14
***Egr1***	transcription factor	2
***Nkx6-2***	transcription factor	5
***Rnaset2***	translation	9
***Rpl30***	translation	5
***Ifitm1***	unknow	10
***Isg12(b)***	unknow	1
***Fbln1***	unknown	1
***LOC365566***	unknown	3
***LOC497841***	unknown	1
***LOC498340***	unknown	3
***LOC499244***	unknown	12
***LOC500804***	unknown	17
***LOC501521***	unknown	1
***LOC501644***	unknown	10
***Ms4a6b***	unknown	2
***Mx1***	virus resistance	1

**Table 3:** Genes differentially expressed across treatments (log2).

*Gene ID*	*Prenatal stress*	*Cumulative stress*	*Ischemic lesion*	*Tactile Stimulation*
***A2m***	-	–2.339601	1.48917	–1.848824
***Adrp***	-	–1.018913	-	-
***Anxa1***	–1.106768	–1.562069	-	–1.407448
***Anxa3***	-	–1.021063	-	–1.006286
***Bmp7***	-	–1.024219	-	-
***C1qa***	-	–1.074996	1.222273	–1.032552
***C1qb***	-	–1.254768	1.127271	–1.090088
***C1r***	-	–1.090324	-	-
***C1s***	-	–1.289453	-	–1.072374
***C2***	-	–1.735695	1.454708	–1.530966
***C4-2***	-	–1.134324	1.125964	-
***Cd48***	-	–1.164392	-	–1.049772
***Cd68***	-	–1.626193	1.357656	–1.220245
Cldn11	-	–1.118507	-	–1.05722
***Col1a2***	–1.610536	–1.830741	-	–1.788933
***Col6a3***	-	–1.261189	-	–1.005407
***Cyba***	-	–1.538457	1.329214	–1.271818
***Dcn***	–1.071648	–1.440197	-	–1.250808
***Emp3***	–1.001066	–1.229681	-	–1.090146
***F2r***	1.520118	-	–1.000434	-
***Fabp7***	-	–1.995661	-	–1.86418
***Ftl1***	–1.275976	–1.479604	1.40322	–1.103008
***Gfap***	–1.55382	–2.16893	1.359457	–2.077125
***Gjb2***	-	–1.285759	-	–1.073373
***Gpx1***	-	–1.040246	-	-
***Gsn***	-	–1.259432	-	–1.143712
***Hmgn1***	-	1.410373	-	–1.236748
***Hspb1***	-	–1.558388	-	–1.440701
***Ifitm1***	–1.496958	–1.746368	-	–1.2543
***Ifitm3***	-	–1.255927	-	–1.232046
Igf2	-	–1.676183	-	-
***Igfbp2***	-	–1.049362	-	-
***Igsf1***	-	–1.238762	-	–1.005989
***Klk6***	-	–1.329943	-	–1.197091
***Lcp1***	-	–1.177579	-	–1.047358
***Lgals1***	–1.070017	–1.264459	-	–1.058962
***Lgals3***	–2.545551	–3.442685	2.278726	–2.881974
***LOC292539***	1.662126	-	-	–1.787299
***LOC365566***	–1.018643	-	-	-
***LOC497841***	-	-	1.040748	-
***LOC498276***	-	–1.475339	1.359406	–1.206185
**LOC498279**	-	–1.234533	-	–1.142965
**LOC498340**	1.201065	-	-	-
***LOC499244***	–1.259298	–1.544845	1.421921	–1.136057
***LOC500804***	–1.286095	–1.505083	1.372513	–1.124729
***LOC501644***	–1.20724	–1.490365	1.192905	–1.100474
***Lr8***	-	–1.243659	1.172925	–1.078479
Mgst1	-	–1.128417	-	–1.075906
***Ms4a6b***	-	–1.016193	-	-
***Mt1a***	-	–1.176589	-	–1.127687
***Ncf1***	-	–1.019811	-	-
***Nkx6-2***	-	–1.032535	-	-
***Npc2***	-	–1.532905	1.136212	–1.312404
***Ogn***	-	–1.053321	-	–1.066816
***Rbp1***	–1.22439	–1.586579	-	–1.386281
***Rnaset2***	-	–1.561604	1.340429	–1.304671
***Rpl30***	-	-	2.114193	-
***Rt1-a2***	-	–1.353097	1.284474	–1.226446
***Rt1-da***	-	–1.203964	-	–1.172782
***S100a4***	–1.932169	–2.97961	1.755045	–2.649376
***S100a6***	–1.323001	–1.790905	-	–1.670722
***S100a11***	–1.515065	–1.835684	-	–1.603393
***S100a13***	-	–1.140052	-	-
***Serping1***	–1.362057	–1.781073	1.330731	–1.512758
***Slc6a5***	-	–1.085427	-	–1.33607
***Smpdl3a***	-	–1.41647	1.114149	–1.152769
***Spp1***	-	–1.72269	1.162702	–1.403692
***Tf***	-	–1.052282	-	-
***Timp1***	-	–1.137261	-	–1.039915
***Trem2***	-	–1.071894	-	-
Txnip	–1.104513	–1.539126	-	–1.403485
***Vim***	–1.715362	–2.347693	1.588672	–2.232288

#### Prenatal stress

Prenatal stress induced gene expression related to essential cellular functions, such as oxidative stress, cell signaling, immune response, and structural maintenance (all statistical significance p<0.05 and fold change≥2; Figure 5A). Among genes differentially expressed between LX and PS+Lx groups the following were up-regulated: *F2r*, *LOC292539*; and down-regulated: *LOC498340*, *LOC365566*, *Lgals3*, *S100a4*, *Vim*, *Col1a2*, *Gfap*, *S100a11*, *Ifitm1*, *Serping1*, *S100a6*, *LOC500804*, *Ftl1*, *LOC499244*, *Rbp1*, *LOC501644*, *Anxa1*, *Txnip*, *Dcn*, *Lgals1*, and *Emp3* (see Table 3).

#### Cumulative prenatal and adult stress

Cumulative stress effects induced expression of genes related to neurotransmission, oxidative stress, cell signaling, apoptosis, growth factors, cell differentiation, transcription factors, translation, DNA repair, immune response, metabolism, and structural maintenance (all statistical significance p<0.05 and fold change≥2; Figure 5B). Among genes differentially expressed between PS+Lx and PS+AS+Lx groups the following was up-regulated: *Hmgn1*; and genes down-regulated were: *Lgals3*, *S100a4*, *Vim*, *A2m*, *Gfap*, *Fabp7*, *S100a11*, *Col1a2*, *S100a6*, *Serping1*, *Ifitm1*, *C2*, *Spp1*, *Igf2*, *Cd68*, *Rbp1*, *Anxa1*, *Rnaset2*, *Hspb1*, *LOC499244*, *Txnip*, *Cyba*, *Npc2*, *LOC500804*, *LOC501644*, *Ftl1*, *LOC498276*, *Dcn*, *Smpdl3a*, *Rt1-a2*, *Klk6*, *C1s*, *Gjb2*, *Lgals1*, *Col6a3*, *Gsn*, *Ifitm3*, *C1qb*, *Lr8*, *Igsf1*, *LOC498279*, *Emp3*, *Rt1-da*, *Lcp1*, *Mt1a*, *S100a13*, *Timp1*, *C4-2*, *Mgst1*, *Cldn11*, *C1r*, *Slc6a5*, *C1qa*, *Trem2*, *Ogn*, *Tf*, *Igfbp2*, *Gpx1*, *Nkx6-2*, *Bmp7*, *Anxa3*, *Ncf1*, *Adrp*, and *Ms4a6b* (see Table 3). Thus, the combination of prenatal and adult stress affected more categories of cell functions than prenatal stress alone.

#### Ischemic lesion

Ischemic lesion induced the expression of genes related to oxidative stress, cell signaling, cell differentiation, translation, immune response, metabolism, and structural maintenance (all statistical significance p<0.05 and fold change≥2; Figure 5C). Among genes differentially expressed between the non-lesion Control group and the Lx group the following were up-regulated: *Lgals3*, *S100a4*, *Vim*, *A2m*, *Rpl30*, *C2*, *LOC499244*, *Ftl1*, *LOC500804*, *Gfap*, *LOC498276*, *Cd68*, *Rnaset2*, *Serping1*, *Cyba*, *Rt1-a2*, *C1qa*, *Lr8*, *Spp1*, *Npc2*, *LOC501644*, *C1qb*, *C4-2*, *Smpdl3a*, *LOC497841*; and *F2r* was down-regulated (see Table 3).

#### Tactile stimulation

TS modulated the expression of genes related to oxidative stress, cell signaling, cell differentiation, translation, immune response, metabolism, structural maintenance, neurotransmission, apoptosis, and DNA repair (all statistical significance p<0.05 and fold change≥2; Figure 5D). Among genes differentially expressed between PA+AS+Lx and PS+AS+TS+LX groups the following were down-regulated: *Hmgn1*, *LOC292539*, *Mt1a*, *Cldn11*, *Hspb1*, *LOC501644*, *C1qa*, *C1qb*, *Ftl1*, *Klk6*, *LOC500804*, *Rt1-a2*, *LOC499244*, *LOC498279*, *Cyba*, *Cd68*, *Npc2*, *Igsf1*, *Rnaset2*, *C2*, *Lgals1*, *Nupr1*, *Gfap*, *Rt1-da*, *Emp3*, *Ifitm3*, *C1s*, *Ifitm1*, *Gsn*, *Lcp1*, *Anxa3*, *Lr8*, *LOC498276*, *Serping1*, *Smpdl3a*, *Slc6a5*, *Timp1*, *S100a11*, *Col6a3*, *Anxa1*, *Cd48*, *Ogn*, *Txnip*, *Spp1*, *S100a6*, *Dcn*, *Mgst1*, *S100a4*, *Rbp1*, *Lgals3*, *Fabp7*, *Gjb2*, *Col1a2*, *A2m*, and *Vim* (see Table 3).

### Cumulative Prenatal Stress and Adult Stress Increased Expression of Hmgn1, and Tactile Stimulation Restores Hmgn1 Expression Levels

Cumulative prenatal stress and adult stress up-regulated *Hmgn1* expression (expression ratio among PS+Lx and PS+AS+Lx in microarray analysis; Figure 5E). TS alleviated AS-induced non-significant up-regulation of *Hmgn1* and decreased its expression level (group PS+AS+Lx vs. group PS+AS+TS+Lx).

### Prenatal Stress Modulates the Expression of Ube2s/LOC365566

Our previous work showed that prenatal stress exposure increased the expression of miR-103 that potentially targets *Ube2s/LOC365566* [6]. Prenatal stress down-regulated *Ube2s,* also known as *LOC365566* or RGD1561498 similar to the ubiquitin-conjugating enzyme *E2s* (expression ratio among Lx and PS+Lx in microarray analysis 9.53624; Figure 5F). TS alleviated PS-induced non-significant down-regulation of *Ube2s/LOC365566* and resulted in an increased expression level (PS+Lx = 8.52; PS+AS+TS+Lx = 9.519).

### Ischemic Lesion Up-regulates Gene Expression

**Figure 6:**
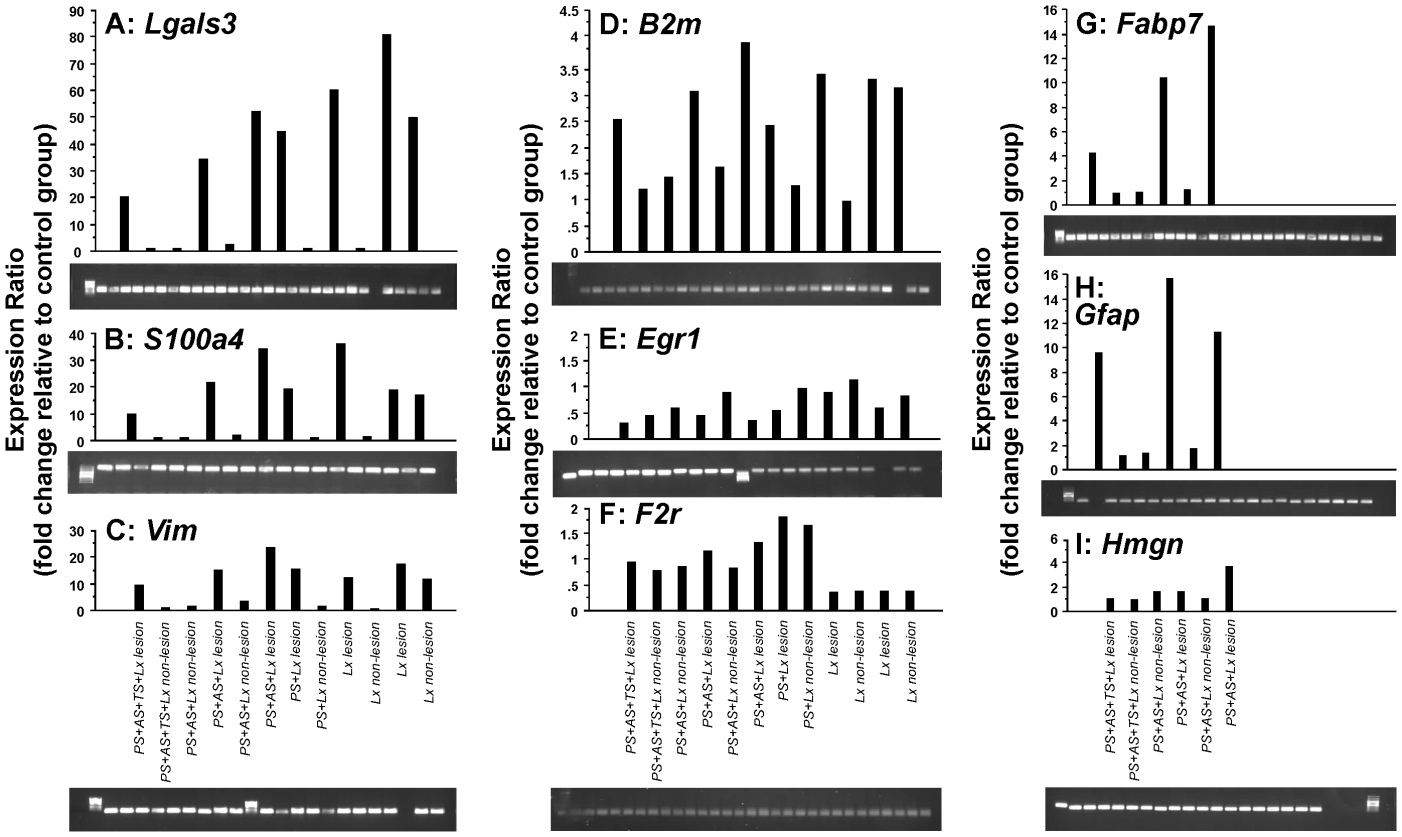
Global gene expression analysis by microarrays was validated by RT-PCR analysis of selected genes. **A,** *Lgals3*; **B,** *S100a4*; **C,** *Vim*; **D,** *B2m*; **E,** *Egr1*; **F,** *F2r*; **G,** *Fabp7*; **H,** *Gfap*; **I,** *Hmgn.* All measurements were compared to naïve controls values. *Gadph* was used as a reference control for calculation of gene expression ratio. PCR-product amplification was confirmed by agarose electrophoresis gels (photographs).

In order to confirm and validate the microarray approach for global gene expression analysis, we performed qRT-PCR analysis of ten genes commonly or differentially regulated. The following genes were analyzed: *Vim*, *S100a4, Lgals3*, *Hmgn1*, *Gst3*, *Gfap*, *Fabp7*, *F2r*, *Egr1*, *B2m*, and *Gadph*. Candidate genes were modulated among groups in the lesion and non-lesion hemispheres when compared with basal expression in non-treated control animals (fold change of control expression ratio; Figure 6). Furthermore, the expression ratios of the above genes in RT-PCR analysis and microarrays were positively correlated. PCR product amplification was confirmed by its detection using agarose electrophoresis gels (Figure 6).

Among all groups, most genes analyzed were up-regulated in both hemispheres when compared with the respective controls (Figure 6). Moreover, the expression ratios were higher for most genes in the lesion hemisphere. Exceptions were *Egr1*, which was down-regulated in the lesion hemisphere in all groups (Figure 6E). Furthermore, *Gst3* showed comparable expression levels in both hemispheres in all groups except that it was down-regulated by TS. In addition, some treatments induced comparable expression levels in the lesion and non-lesion hemispheres, including *F2r* (ischemic lesion) and *Hmgn* (TS).

## Discussion

The present study shows that early life experiences exert life-long influences on behaviour and recovery following ischemic motor cortex lesion. These changes occur in association with distinct profiles in the brain transcriptome. Cumulative PS and AS interacted with genes related to growth factors and transcription factors, which were not affected by PS or lesion alone. Furthermore, we used TS to attenuate the impact of an adverse early environment and promote recovery from ischemic lesion. TS in PS+AS animals reverted changes in gene expression involving growth factors and transcription factors that were induced by stress and lesion. These findings support the notion that programming by adverse or favorable experiences can occur throughout life and modulates behaviour and recovery following brain injury.

### Prenatal Stress Programs Motor Function and Recovery from Stroke

Prenatal stress can delay development, alter HPA axis function and behavioural outcomes [39], [10], [40]–[41], [5]. Here we show that prenatal stress affects skilled movement in adulthood. Our findings are in accordance with previous reports of altered skilled hindlimb use in prenatally stressed rats [42] and skilled reaching impairments in marmosets antenatally treated with dexamethasone [43]. Prenatal stress may cause long-lasting changes in fine motor control based on abnormal motor efferent pathway formation or changes in central dopaminergic systems [44]–[45].

Notably, prenatal stress exaggerated the response to recurrent stress in adulthood as illustrated by quantitative and qualitative movement performance. These findings agree with reports of fetal HPA axis activity programming by altered density of hippocampal glucocorticoid receptors and mineralocorticoid receptors [46], [3] and exaggerated responses to adversity later in life [47], thus altering stroke motor recovery.

Prenatal stress aggravated functional loss after focal ischemic lesion similar to the effects previously shown for stress in adulthood [11]–[12]. The functional impairments associated with stress, however, were not necessarily accompanied by corticosterone elevation. The presence of movement impairments in the absence of elevated corticosterone levels suggests that stress-induced motor impairments might be independent of corticosterone [28], [48]. The causes of stress-induced limitation in motor recovery may be linked to excitotoxic processes (Madrigal et al., 2003), reduced expression of anti-apoptotic [49] and neurotrophic factors [50]–[51]. All of these may limit cell survival and plastic rearrangements [52]–[53] and impede behavioural compensation and neuronal plasticity [54]–[55]. Moreover, prenatal stress modulates neuronal development in frontal cortex [56]–[57], thus potentially involving motor cortex and the corticospinal tract to alter fine motor control.

### Tactile Stimulation Promotes Recovery from Stroke

The present observations indicate that TS in rats protects against stress-induced aggravation of lesion-induced fore- and hindlimb motor loss. Accordingly, body massage has been a prominent therapy to reduce the effects of stress and relieve associated affective states in humans [18], [17] and was considered as a potential complementary therapy in stroke patients [19]–[20]. TS was shown to serve as an equivalent to massage therapy in animals by promoting recovery from brain damage in newborn and adult rats [16], [58].

The preserved qualitative movement trajectories in lesion rats confirm the notion that TS has neuroprotective effects [28], allowing the damaged brain to maintain the integrity of motor pathways. TS seems to particularly promote genuine recovery of skilled reaching movements after brain damage [28], [59]. These findings suggest that TS is potent enough to offset the effects of prenatal stress and the consequences of exaggerated stress responses on stroke outcome. Considering the conservative scoring methods of single pellet reaching applied to daily test sessions [33], the present improvements represent robust and functionally meaningful outcomes. Our observations agree with earlier reports showing that TS can reduce the adversity of maternal deprivation or preterm birth in rodents and humans [22]–[24], [60]. Morphological correlates of these effects include cortical rearrangement and enhanced synaptogenesis in pyramidal cells in adjacent cortical areas [15], [25], [16]. The neuroprotective effects of TS are supported by findings that maternal licking and grooming or TS can alleviate inflammatory functions [61]. In a different context, TS may represent an aversive stimulus due to its need for animal handling and maternal separation [62]. Thus, the present study applied TS to adult offspring with handling controls as a comparison.

The positive effects observed on stroke motor recovery may in part be explained by neurotrophic factor regulation. TS-induced neurotrophic factor modulation may mediate the sparing of motor function [63]. Accordingly, TS may affect insulin-like growth factor-1 (IGF-1), a protein linked to a signaling pathway involved in aging and neurotrophic functions [64]–[65]. Further, TS was shown to affect cellular metabolism, levels of stress hormones (Kassahn et al., 2009) and to activate 5-HT systems and initiate intracellular signaling pathways that regulate glucocorticoid receptor (GR) transcription [63]. Our findings indicate that TS can help adjust abnormal HPA axis activity, behavioural and transcriptomic programming effects of perinatal adverse experience at any time in life.

### Adverse and Positive Experiences are Reflected in Motor Cortex Transcriptomic Profiles

Differential mRNA profiles among groups suggest a molecular basis for early life events to sculpt later stress response, behaviour and recovery from brain injury. Cumulative effects of stress increased the diversity of pathways induced by prenatal stress. Ischemic lesions significantly up-regulated gene expression. By contrast, the beneficial effects of TS were linked to down-regulated gene expression patterns. These contrasting transcriptomic patterns may have critical implications for the phenotypic effects of adversity versus positive experiences.

Prenatal stress, cumulative stress, ischemia, and TS modulated the expression of several genes specifically related to nervous system function. Among them, *Vim* (vimentin) encoding a protein involved in structural maintenance, was down-regulated in most treatments, but up-regulated by ischemic lesion. GR-mediated signaling reduces *Vim* expression, which is possibly linked to visual function [66] and astrocyte morphology [67]. Increased VIM expression was observed in the post-ischemic kidney [68], which confirms the present findings. Moreover, *Gfap* (glial fibrillary acidic protein) was also down-regulated in all groups, but up-regulated by ischemia. *Gfap* is related to a differential phenotype in astrocytes and neural stem cells in the aging brain [69]. These findings indicate that early and late lifespan adversity may modulate structural neuronal maintenance and connectivity.

Gene expression changes also involved proteins that are vital to neurological function and cerebral disorders. *S100a4* related to cell signaling was down-regulated in all treatments, but up-regulated by ischemia. S100A4 is involved in axon and dendrite elongation and astrocyte migration [70]. Prenatal stress also down-regulated *S100a6* encoding a calcium-binding protein related to cell signaling and ion transport in astrocytes and involved in Alzheimer's disease, Down syndrome and amyotrophic lateral sclerosis [71]. Cumulative stress and ischemia, on the other hand, modulated *A2m* expression, a gene encoding a protease inhibitor and cytokine transporter. A2M is implicated in Alzheimer’s disease (AD) and mediates the clearance and degradation of β-amyloid deposits [72]. Cumulative stress also down-regulated gene expression for FABP7, which plays a role in developing rat cortex [73] and for CLDN11, a major component of CNS myelin important to oligodendrocyte proliferation and migration [74]. SLC6A5 is a glycine neurotransmitter transporter implicated in hyperekplexia, a neuromotor disorder [75]–[76]. *Slc6a5* was down-regulated by cumulative stress and TS. Cumulative stress decreased levels of *Igfbp2* (insulin-like growth factor binding protein 2), which has also been reported in obese humans [77]. In summary, our findings suggest that early and late life adversity may potentially have profound consequences on neuronal and glial function.

Among the pathways altered by prenatal stress was HMGN1 (high-mobility group nucleosome binding domain 1), a nuclear protein involved in compaction of chromatin [78]–[79], histone modifications [80], DNA repair [81] and epigenetic regulation of neurodevelopmental diseases [82], such as Down syndrome [83], [82]. Interestingly, TS down-regulated *Hmgn1* in motor cortex and offset the effects of cumulative stress on stroke recovery. Notably, HMGN1 can negatively regulate the expression of methyl-CpG-binding protein 2 (MeCP2), a DNA-binding protein that reinforces DNA methylation and promotes formation of inactive chromatin. Changes in HMGN1 abundance and deposition of MeCP2 in the genome may affect neurological functions [82]. Depletion of MeCP2 in neurons induces a functional deficit in neuronal activity and plasticity [84]–[86], thus exaggerating the loss of behavioural capacity resulting from stroke. MeCP2 depletion may be linked to angiogenesis and reperfusion after stroke [87]. A second pathway investigated here involved *Ube2s/LOC365566*, a gene targeted by miR-103. Prenatal stress was shown to increase miR-103 expression [6], which supports the present finding of *Ube2s/LOC365566* down-regulation. The functional consequences of this association still remain to be explored.

The transcriptomic changes found here are arguably associated with epigenetic regulation that alters disease outcomes, such as stroke [88]–[89], [6]. Even short periods of stress in adulthood induce miRNA-mediated transcriptomic programming of the motor system [90]. Such epigenetic processes linked to prenatal stress may influence brain development and stress response with lifetime consequences on behaviour.

## Conclusion

The present observations suggest that stress during the second half of pregnancy in the rat occurs during a critical phase of fetal sensorimotor and stress response programming to affect motor function and neurological outcomes after focal ischemic infarct later in life. By contrast, enriched multimodal experiences, such as TS, may effectively intervene to offset the consequences of even remote adverse experience that occurred early in life.
