# Correction: Lifetime Stress Cumulatively Programs Brain Transcriptome and Impedes Stroke Recovery: Benefit of Sensory Stimulation

**DOI:** 10.1371/journal.pone.0102489

**Published:** 2014-07-07

**Authors:** The PLOS ONE Staff

There is an error in the key in Figure 5. The letters “MT” should be replaced with the letters “TS”. The line “PS + AS + MT + Lx” should read “PS + AS + TS + Lx”. Please see the corrected Figure 5 below.

**Figure 1:**
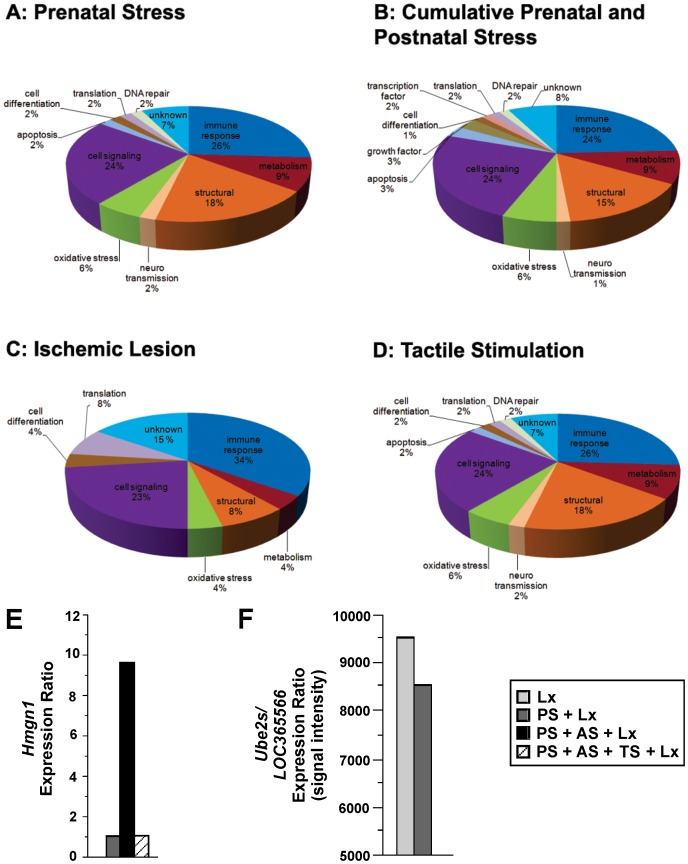
10.1371/journal.pone.0092130.g005
